# Thermodynamic Tuning of Mg-Based Hydrogen Storage Alloys: A Review

**DOI:** 10.3390/ma6104654

**Published:** 2013-10-18

**Authors:** Min Zhu, Yanshan Lu, Liuzhang Ouyang, Hui Wang

**Affiliations:** 1 School of Materials Science and Engineering, South China University of Technology, Guangzhou 510641, China; E-Mails: yanshan.lu@mail.scut.edu.cn (Y.L.); meouyang@scut.edu.cn (L.O.); mehwang@scut.edu.cn (H.W.); 2 Key Laboratory of Advanced Energy Storage Materials of Guangdong Province, Guangzhou 510641, China

**Keywords:** Mg-based hydrogen storage alloys, thermodynamics, alloying, nanostructuring, changing reaction pathway

## Abstract

Mg-based hydrides are one of the most promising hydrogen storage materials because of their relatively high storage capacity, abundance, and low cost. However, slow kinetics and stable thermodynamics hinder their practical application. In contrast to the substantial progress in the enhancement of the hydrogenation/dehydrogenation kinetics, thermodynamic tuning is still a great challenge for Mg-based alloys. At present, the main strategies to alter the thermodynamics of Mg/MgH_2_ are alloying, nanostructuring, and changing the reaction pathway. Using these approaches, thermodynamic tuning has been achieved to some extent, but it is still far from that required for practical application. In this article, we summarize the advantages and disadvantages of these strategies. Based on the current progress, finding reversible systems with high hydrogen capacity and effectively tailored reaction enthalpy offers a promising route for tuning the thermodynamics of Mg-based hydrogen storage alloys.

## 1. Introduction

Modern civilization would not be sustainable without sufficient energy and a clean environment. However, severe challenges will arise from the shortage of fossil energy resources (coal, oil, and natural gas) and environmental pollution, owing to massive and long-term use of fossil fuels. Thus, it is very important to develop clean and renewable energy sources to replace fossil fuels.

Hydrogen is considered to be one of the most promising energy carriers for the future, because it is abundant, has high energy density (142 MJ/kg, three times higher than that of gasoline), and its combustion product is environmentally benign water [1]. However, hydrogen exists as a gas under atmospheric conditions and is highly flammable, explosive, and diffusible. Thus, storing hydrogen in a safe and efficient way is very important, but difficult, for hydrogen to be used as an energy source.

Hydrogen storage methods can be classified into three types: high-pressure gas storage, low-temperature liquid storage, and solid-state storage. Although compressed hydrogen gas technology is relatively mature, the energy content per unit volume is only 4.4 MJ/L at pressures as high as 70 MPa, which is much less than that of gasoline (31.6 MJ/L) [2]. In addition, it has other drawbacks such as risks due to the very high pressures and large energy consumption during compression. Liquid hydrogen storage (8.4 MJ/L) has almost twice the energy content of hydrogen gas [2]. However, hydrogen liquefaction requires cooling to −252 °C, which is expensive and there are problems with inevitable evaporative loss [2,3]. Solid-state hydrogen storage involves storing hydrogen in solid-state materials through physical or chemical absorption, and is considered as the most promising method owing to its high energy density and safety. Currently, solid-state materials such as carbon materials, zeolites, and metal organic frameworks (MOFs) adsorb molecular hydrogen mainly through physisorption, while metal hydrides, complex hydrides, and chemical hydrides (e.g., ammonia borane) store atomic or ionic hydrogen by chemical bonding.

**Figure 1:**
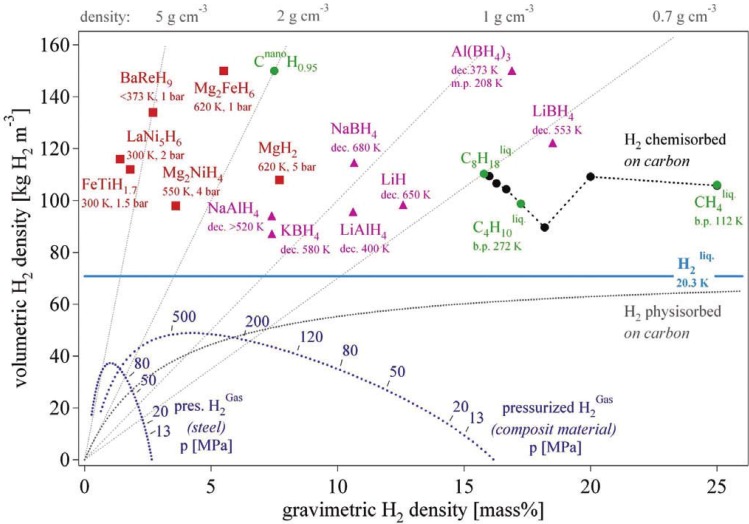
Volumetric and gravimetric hydrogen density of some hydrides (Reprinted with permission from [4], Copyright 2003 Elsevier).

Figure 1 shows the volumetric *versus* gravimetric hydrogen density of the various hydrogen storage methods [4]. Taking into account storage density, energy consumption, and safety, metal hydrides and complex hydrides are the most promising materials for solid-state hydrogen storage. Metal hydrides usually consist of an element A (e.g., Ti, Zr, Mg, and Re) and an element B (e.g., Fe, Co, Ni, and Cu) that tend to form a stable and an unstable hydride, respectively. Based on the atomic ratio of A to B, metal hydrides can be classified as AB_5_, AB_3_, AB_2_, AB type compounds.

LaNi_5_ has a CaCu_5_ type structure and is a typical example of an AB_5_ type alloy [5]. At ambient temperature, LaNi_5_H_6_ forms upon hydrogenation with a theoretical hydrogen capacity of about 1.4 wt % and a formation enthalpy of −30.2 kJ/mol. A successful application of the AB_5_ type alloys has been achieved using the negative electrode in Ni/MH batteries. Substitution of La in LaNi_5_ with Mm (mischmetal) and Ni with Mn, Co, and Al is beneficial for improving the cycle-life and reducing the cost. One of the AB_5_ type alloys with excellent properties is MmNi_3.55_Al_0.4_Mn_0.3_Co_0.75_ [6]. The AB_2_ and AB type alloys, which have Laves and B2 structures, and hydrogen storage capacities of about 2.0 wt % and 1.8 wt %, respectively. These alloys have also been used in practical applications such as rechargeable batteries, hydrogen sources for fuel, and hybrid compressed hydrogen cylinders [7,8].

To increase the hydrogen storage capacity, Mg is added into the AB_5_ alloys, and AB_3_ type (PuNi_3_ or CeNi_3_ structure) alloys are obtained. The AB_3_ structure contains a long-range stacking arrangement of which one-third is AB_5_-like and two-thirds is AB_2_-like. Chen *et al.* [9] prepared LaNi_3_ and CaNi_3_ using a powder metallurgy method. Composition optimization is very important for achieving excellent electrochemical performance, including capacity, rate capability, and cycle-life. Kohno *et al.* [10] reported that partially substituting Ni by Al, Fe, Mn, Si, Sn, and Cu increases the discharge capacity of La(Ni_0.9_M_0.l_)_3_ alloy electrodes at room temperature. By partial substitution, AB_3_ alloys, such as LaCaMgNi_9_, CaTiMgNi_9_, LaCaMgNi_6_Al_3_, and LaCaMgNi_6_Mn_3_, were also synthesized. All of these alloys can be easily activated at room temperature under a hydrogen pressure of 3.3 MPa and can absorb/desorb 1.8 wt % hydrogen [9]. The theoretical discharge capacity of the AB_3_ alloy electrode LaCaMgNi_9_ is 484 mAh/g, which is 30% higher than that of the AB_5_ alloys (372 mAh/g) [11]. Peng *et al.* [12] developed a Ml_0.7_Mg_0.3_Ni_3.2_ (Ml denotes La-rich mischmetal) hydrogen storage alloy by induction melting and found that the alloy had a multi-phase microstructure containing (MlMg)Ni_3_, (MlMg)Ni_2_, and MlNi_5_ phases with a maximum hydrogen storage capacity of 1.7 wt % at room temperature. They also found that the grain size has a large effect on the hydrogen storage capacity of AB_3_-base Ml–Mg–Ni multi-phase alloys. The hydrogen storage capacity decreases with decreasing grain size, which is due to poor hydrogen storage in the grain boundary region [13].

The above mentioned metal hydrides have suitable hydrogen sorption thermodynamics and fast kinetics, which means that hydrogen sorption can take place with a fast rate at suitable temperatures and hydrogen pressure conditions. However, they all have low gravimetric hydrogen storage density and do not satisfy the application requirements as energy sources, in particular the goal for the storage of at least 5.5 wt % by the year 2017 set for onboard automotive hydrogen storage systems by the United States Department of Energy (DOE) [14]. Mg-based hydrides have relatively high storage capacity (theoretically 7.6 wt % for MgH_2_), are abundant and inexpensive, and are considered to be the most promising metallic hydrogen storage materials. However, poor kinetics and high operating temperatures (up to about 300 °C at 1 atm hydrogen pressure) hinder their practical application. Thus, tuning the kinetics and thermodynamics of Mg-based hydrides is the key issue to overcome the problems limiting their practical application. Significant progress has been made in improving the hydrogenation/dehydrogenation kinetics of Mg-based alloys, which will be briefly reviewed in the following sections. However, tuning of the thermodynamic properties is still a great challenge, and we will review the progress in detail.

## 2. Thermodynamic and Kinetic Characteristics of Mg-Based Hydrogen Storage Alloys

Pure Mg has a hexagonal crystal structure, and it can react reversibly with hydrogen to form MgH_2_. As the hydrogen pressure increases, the crystal structure of MgH_2_ changes to tetragonal β-MgH_2_. At ambient temperature and high hydrogen pressure (GPa), β-MgH_2_ transforms to orthorhombic γ-MgH_2_. γ-MgH_2_ is a metastable phase and decomposes to the stable β-MgH_2_ phase at temperatures above 350 °C. Table 1 shows the crystal structure data of Mg, β-MgH_2_, and γ-MgH_2_.

**Table 1:** Crystallographic data of Mg, β-MgH_2_, and γ-MgH_2_.

Phase	Space group	Lattice parameters (Å)	Prototype
Mg	*P*6_3_*/mmc*	*a* = 3.2094, *c* = 5.2112 (PDF#35-0821)	Mg
β-MgH_2_	*P*4_2_*/mnm*	*a* = 4.5170, *c* = 3.0205 (PDF#12-0697)	Rutile TiO_2_
γ-MgH_2_	*Pbcn*	*a* = 4.5300, *b* = 5.4400, *c* = 4.9300 (PDF#35-1184)	α-PbO_2_

Because of the strong ionic characteristics of the Mg–H bond, the desorption temperature of MgH_2_ is relatively high. The enthalpy can be used to characterize the strength of metal-H bonds, and the enthalpy and entropy can be calculated by the van’t Hoff equation according to the equilibrium hydrogen pressure and the corresponding temperature in the pressure-composition isotherm (PCI) of hydrogen absorption/desorption. The measured thermodynamic data of MgH_2_ from different works are shown in Table 2. The desorption enthalpy of MgH_2_ is much larger than practical requirements for metal hydrides of 20–40 kJ/mol. Corresponding to its thermodynamic characteristics, the equilibrium hydrogen desorption temperature is 289 °C under 1 atm hydrogen pressure. Thus, from a thermodynamic point of view, Mg/MgH_2_ is unsuitable for practical application as a hydrogen storage material unless the ΔH of Mg-based alloys can be decreased. ΔS is another important thermodynamic value for hydrogen desorption. The entropy has been considered to be a constant value of about 130 J/(mol·K), however, recent studies have shown that ΔS of the desorption process is variable [15,16]. Therefore, increasing the entropy would be a useful way of lowering the operating temperature if ΔH did not significantly change.

**Table 2:** ΔH and ΔS for MgH_2_ decomposition in different works.

Different works	ΔH (kJ/mol)	ΔS [J/(mol·K)]
Stampfer [17]	74.4 ± 0.3	135.1 ± 1.9
Reilly [18]	77.4 ± 4.2	138.3 ± 2.9
Pedersen [19]	70	126
Friedlmeier [20]	74.3 ± 0.5	136 ± 1
Shao [21]	75.0	135.6
Bardhan [22]	75	130

**Figure 2:**
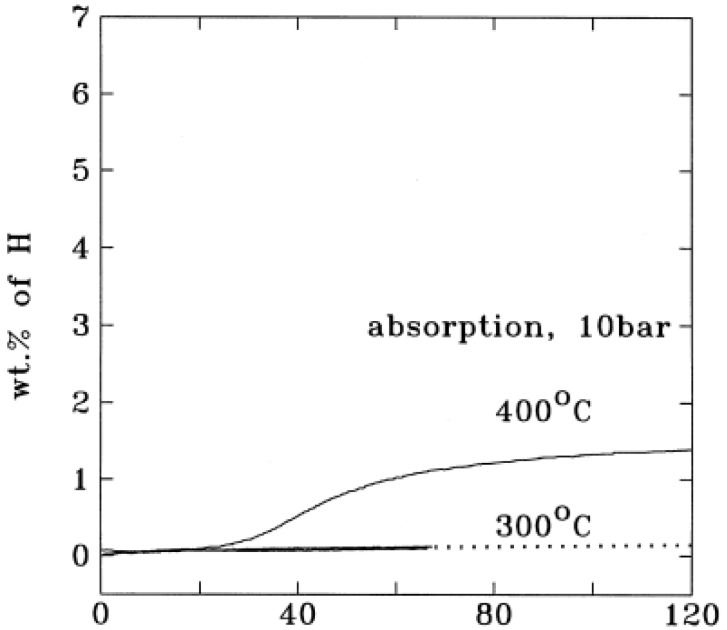
Hydrogen absorption by a conventional, non-catalyzed magnesium powder (Reprinted with permission from [23]. Copyright 1999 Elsevier).

**Figure 3:**
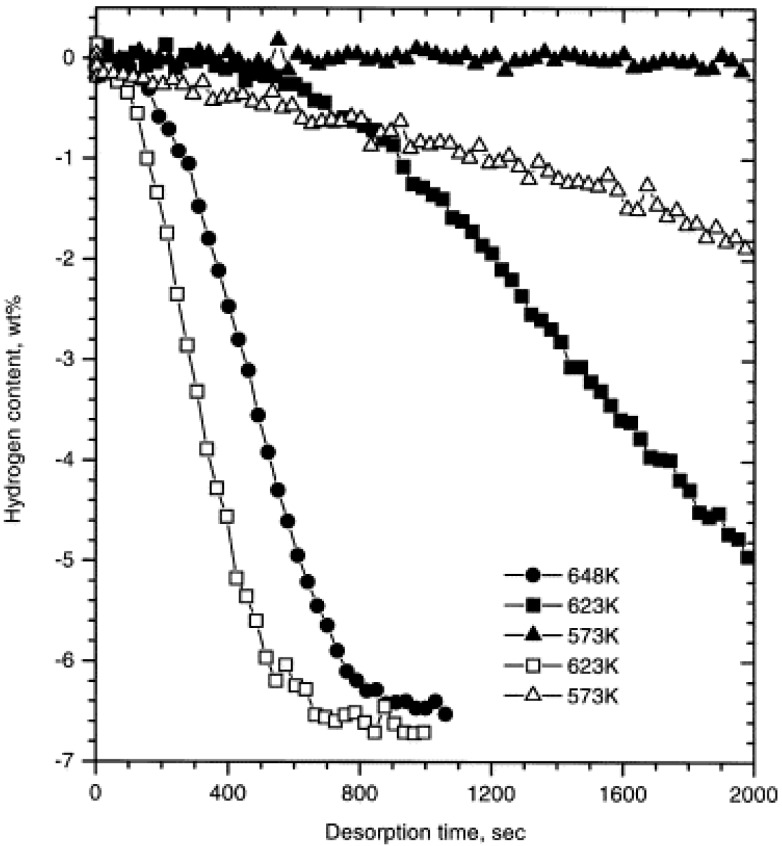
Hydrogen desorption curves of unmilled (solid symbols) and ball-milled (hollow symbols) MgH_2_ under a hydrogen pressure of 0.015 MPa (Reprinted with permission from [24]. Copyright 1999 Elsevier).

Apart from the above mentioned thermodynamic restrictions, the poor kinetics of Mg-based hydrides also result in its high operating temperature and limit its practical use. Figure 2 shows the hydrogenation kinetic curves of conventional Mg powder. The hydrogen absorption capacity was almost negligible when heated up to 300 °C. Even when the powder was heated to 400 °C, the hydrogen absorption capacity was less than 2 wt % after 2 h [23]. For the dehydrogenation of unmilled MgH2 (Figure 3), there is no obvious desorption at 573 K within 2000 s. Even at 623 K, it takes more than 3000 s to completely desorb. The high activation energy (Δ*E*) for hydrogen desorption from unmilled MgH_2_ (about 156 kJ/mol) is a clear indication of the poor kinetics [24].

There are several reasons for the sluggish hydrogenation kinetics: (1) a layer of MgO or Mg(OH)_2_ can easily form on the surface and inhibit the dissociation of molecular H_2_ on the surface and diffusion of atomic H into the bulk; and (2) the diffusion of H is extremely difficult after a MgH_2_ layer forms on the surface of Mg because the H_2_ diffusion coefficient in MgH_2_ (1.5 × 10^−^^16^ m^2^/s) is considerably smaller than that in Mg (4 × 10^−^^13^ m^2^/s) [25]. The main reasons for the sluggish dehydrogenation kinetics are: (1) the difficulty of breaking the Mg–H bond; (2) the low diffusion rate of H in MgH_2_; (3) the high energy required for the nucleation of Mg on the surface of MgH_2_; and (4) recombination of hydrogen atoms to form the hydrogen molecule on the Mg surface [26,27,28].

## 3. Progress in Improving the Hydrogen Absorption/Desorption Kinetics of Mg-Based Alloys

To accelerate the hydrogen absorption/desorption kinetics of Mg-based hydrides, tremendous efforts have been devoted to develop methods of catalyzing, nanostructuring, and forming Mg-based composites. Significant progress has been achieved through those efforts.

Transition metals and metal oxides have proved to be effective catalysts for hydrogen absorption/desorption of Mg-based hydrides. Liang *et al.* [29] mixed MgH_2_ with the 3d elements Ti, V, Mn, Fe, and Ni by mechanical milling. All of the additives drastically decreased the activation energy of hydrogen desorption of MgH_2_. The MgH_2_–V composite showed the most rapid desorption kinetics, and its activation energy of 62.3 kJ/mol is much smaller than that of ball-milled pure MgH_2_ (120 kJ/mol [24]), while the composite containing Ti exhibited the most rapid absorption kinetics. Bormann *et al.* [30] produced nanocrystalline MgH_2_/Me*_x_*O*_y_* (Me*_x_*O*_y_* = Sc_2_O_3_, TiO_2_, V_2_O_5_, Cr_2_O_3_, Mn_2_O_3_, Fe_3_O_4_, CuO, Al_2_O_3_, and SiO_2_) powders via high-energy ball milling. Compared with the pure nanocrystalline materials, some oxides lead to an enormous catalytic acceleration of hydrogen sorption. Cr_2_O_3_ gave the fastest hydrogen absorption, and the composite material containing Fe_3_O_4_ showed the fastest desorption kinetics. Later, they found that the catalytic effect of Nb_2_O_5_ was superior for the hydrogen sorption reaction of magnesium compared with other metal and oxide catalysts, with absorption and desorption of 7 wt % hydrogen possible within 60 s and 130 s at 300 °C, respectively [31]. The additives catalyze the reaction by promoting the recombination of hydrogen atoms to form the hydrogen molecule. The catalytic activity of the additives is influenced by four distinct physico-thermodynamic properties: a high number of structural defects, a low stability of the compound, a high valence state of the transition-metal ion within the compound, and a high affinity of the transition-metal ion for hydrogen [32]. However, the thermodynamics of MgH_2_ are obviously not changed by adding the catalysts.

Nanostructuring is an efficient way of improving the kinetics of Mg-based alloys. Ball milling is a common method to prepare nanocrystalline Mg-based hydrides, and significantly increases the hydrogen uptake/release rates of Mg/MgH_2_ because it destroys the oxide layers on the surface of Mg, introduces a large number of defects, and decreases the diffusion lengths of H. Ball-milled MgH_2_ completely desorbed at 350 °C after 700 s, and reabsorbed in 750 s at 300 °C [24]. To overcome the drawback of the high oxidation sensitivity of Mg-based materials, especially at the nanoscale in air, Jeon *et al.* [33] prepared an air-stable composite material consisting of metallic Mg nanocrystals (NCs) in a gas-barrier polymer matrix that was capable of both storing a high density of hydrogen (up to 6 wt % of Mg, 4 wt % for the composite) and rapid kinetics (loading in <30 min at 200 °C). Moreover, nanostructuring of Mg results in rapid storage kinetics without using expensive heavy-metal catalysts.

*In situ* formation of a nanocomposite, in which catalyst phases are involved, is another efficient way of improving the kinetics of Mg-based alloys. Ouyang *et al.* prepared a series of Mg_3_RE (RE = La [34], Pr [35], Nd [36], and mischmetals [37]) alloys by induction melting. All of the alloys could absorb hydrogen at room temperature with rapid hydrogenation/dehydrogenation kinetics. After the first hydrogenation, MgH_2_ and REH*_x_* composites were formed, although REH*_x_* was almost unchanged in the subsequent hydrogenation/dehydrogenation cycles. The nanometer-sized REH*_x_* formed *in situ* during the activation process showed good catalytic properties for increasing the kinetics. The kinetics could be further accelerated by alloying Mg_3_RE with transition metals such as Ni and Co [34,35,38]. Recently, Liu *et al.* [39] prepared the Mg_91.9_Ni_4.3_Y_3.8_ alloy composed of a large amount of long-period stacking ordered (LPSO) phases. The alloy showed excellent dehydrogenation kinetics and could release about 5 wt % hydrogen at 300 °C within 200 s. At the first hydrogenation reaction, the LPSO phases transformed into MgH_2_, Mg_2_NiH*_x_*, and YH*_x_*. Although the transformations could not reversibly take place during the subsequent hydrogenation reaction, the highly dispersed and nanostructured composite (MgH_2_ + Mg_2_NiH*_x_* and YH*_x_*) remained after the hydrogenation/dehydrogenation cycles, which effectively promotes the dehydrogenation performance of the alloy. In addition, YH*_x_* and Mg_2_NiH*_x_* can also act as a catalyst for the hydrogenation/dehydrogenation of the alloy.

The hydrogen storage properties of Mg-based alloys have also been improved by *in situ* formation of nanocomposites with the melt-spun method. Under condition of rapid solidification, Mg–Ni–RE (RE = rare earth metal or Y) alloys can be easily amorphized. Spassov et al. [40,41] prepared a series of nanocrystalline and nano-amorphous Mg–Ni–RE alloys with good hydrogen storage properties by rapid quenching. For example, the as-quenched Mg_75_Ni_20_Mm_5_ alloy could absorb 4.0 wt % H_2_ at 25 °C in 100 min. The Mg_75_Ni_20_Mm_5_ alloy consisted of nanocrystals embedded in an amorphous phase, and the amorphous phase around the nanocrystals gave hydrogen easier access to the nanograins, avoiding the long-range diffusion of hydrogen through an already formed hydride, which is often the slowest step of absorption. Lin *et al.* [42] reported that after only one activation cycle under H_2_ (4 MPa) at 300 °C, melt-spun Mg_3_LaNi_0.1_ alloy could absorb 2.7 wt % hydrogen at room temperature within 3 min, and the minimum hydrogen desorption temperature was 224 °C, which is 33 °C lower than that of the Mg_3_LaNi_0.1_ melt. This improvement was attributed to the catalytic role of the *in situ* formed nanocrystalline Mg_2_Ni and LaH_2_.

**Figure 4:**
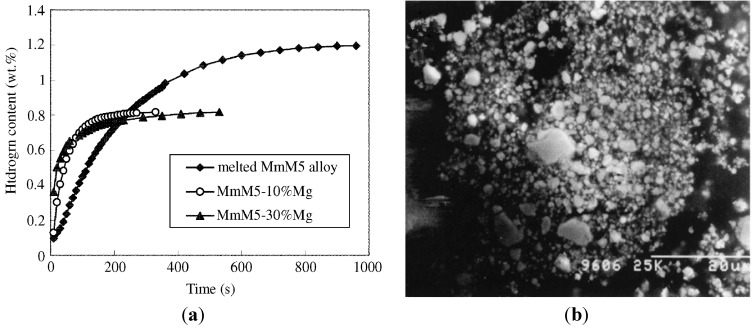
(**a**) Hydriding kinetic curves for melted MmM_5_ alloy and nano-phase composite of compositions MmM_5_–10% Mg and MmM_5_–30% Mg measured under isobaric conditions at 333 K (Reprinted with permission from [43]. Copyright 2002 Elsevier); (**b**) SEM micrograph of MmM_5_–10% Mg powder mixture milled for 20 h (Reprinted with permission from [44]. Copyright 2003 Springer).

Forming Mg-based composites with other hydrogen storage alloys can enhance the hydriding process of Mg, which is due to the key role played by the interphase boundary in the interaction between the different components of the composite. Zhu *et al.* [43] prepared Mg-MmM_5_ (LaNi_5_-based RE alloys) nano-phase composite hydrogen storage alloys by mechanical alloying. Figure 4a shows that in the MmM_5_–10% Mg and MmM_5_–30% Mg systems, nanocrystalline Mg can quickly absorb hydrogen at room temperature. The hydrogen absorption kinetic curves were fitted using various rate equations to reveal the mechanism of the hydriding reaction process. For the nano-phase composite, the hydriding reaction was in agreement with the rate equation ln[α/(1 − α)] = *k*(*t* − *t_c_*) of an auto-catalysis process. Figure 4b is a secondary electron image of MmM_5_–10% Mg powder obtained by milling for 20 h. After the long milling time, the brittle MmM_5_ component was very fine and bonded to softer Mg, forming composite particles that contain a very high density of interfaces [44]. Gross *et al.* [45] produced La_2_Mg_17_–LaNi_5_ composites by mechanically milling. The kinetics of the composites are superior to those of pure La_2_Mg_17_ due to the composite consisting of a complex porous agglomeration of three phases: Mg_2_Ni (~1 μm), La (~100 nm), and Mg. The absorption and desorption kinetics of Mg are catalytically enhanced by the intimate contact with Mg_2_Ni, and to a lesser extent by the La phase.

## 4. Strategies for Tuning the Thermodynamics of Mg-Based Alloys

As described above, the hydrogen absorption/desorption kinetics have been improved substantially for Mg-based alloys. However, the high thermodynamic stability of MgH_2_ is still a big hurdle for lowering the hydrogen desorption temperature of Mg-based hydrides. Effective attempts, such as alloying, nanostructuring, and changing the hydriding/dehydriding reaction route, have been made to tune the thermodynamics of Mg-based alloys.

### 4.1. Alloying

Mg forms the stable hydride MgH_2_ with negative enthalpy (Δ*H* = −75 kJ/mol). If Mg alloys are made with a hydride non-forming element, the hydriding reaction enthalpy can be decreased. Therefore, alloying is a traditional and effective strategy for altering the thermodynamics of Mg-based alloys.

One of the typical examples is Mg_2_Ni (Ni is the hydride non-forming element), which can react with H_2_ to form Mg_2_NiH_4_. The formation enthalpy of Mg_2_NiH_4_ (−64.5 kJ/mol) is lower than that of MgH_2_ [18]. Morinaga *et al.* [46] found that hydrogen interacted more strongly with Ni atoms rather than Mg atoms in Mg_2_NiH_4_. This Ni–H interaction in Mg_2_NiH_4_ is much weaker than the Mg–H interaction in pure MgH_2_, and leads to a lower formation enthalpy of Mg_2_NiH_4_. However, Mg_2_NiH_4_ has a lower hydrogen storage capacity than MgH_2_. Similar to the Mg–Ni system, Mg and Cu can form the Mg_2_Cu alloy. During hydrogenation, Mg_2_Cu decomposes to MgH_2_ and MgCu_2_ [47], and the equilibrium temperature for 1 bar hydrogen pressure is reduced to about 240 °C. Unfortunately, this reaction is irreversible.

Mg and Fe are immiscible, but a ternary hydride Mg_2_FeH_6_ can be formed in the presence of hydrogen [48]. Mg_2_FeH_6_ is very attractive as a hydrogen storage material because of its high volumetric and gravimetric hydrogen capacity, up to 150 kg/m^3^ and 5.5 wt %, respectively. However, the formation enthalpy of Mg_2_FeH_6_ (−77.4 kJ/mol [49]) is higher than that of MgH_2_. In addition, preparation of Mg_2_FeH_6_ is difficult as Mg and Fe do not form any intermetallic compounds. Mg and Co can react with H_2_ under certain conditions to form Mg_2_CoH_5_. The gravimetric and volume density of Mg_2_CoH_5_ are 4.5 wt % and 100 kg/m^3^, respectively. However, the dissociation heat of Mg_2_CoH_5_ is 86 kJ/mol [50], and Mg_2_CoH_5_ is not easy to synthesize because Mg_2_Co does not exist. Ti also does not form an alloy with Mg under conventional conditions. Kyoi *et al.* [51] prepared a Mg_7_TiH*_x_* hydride by reacting MgH_2_ with TiH_1.9_ at 8 GPa and 873 K in a high-pressure anvil cell. Mg_7_TiH*_x_* decomposes into Mg and TiH_1.9_ with the release of 4.7 wt % hydrogen, and the desorption temperature is around 605 K, which is 130 K lower than that of MgH_2_.

**Figure 5:**
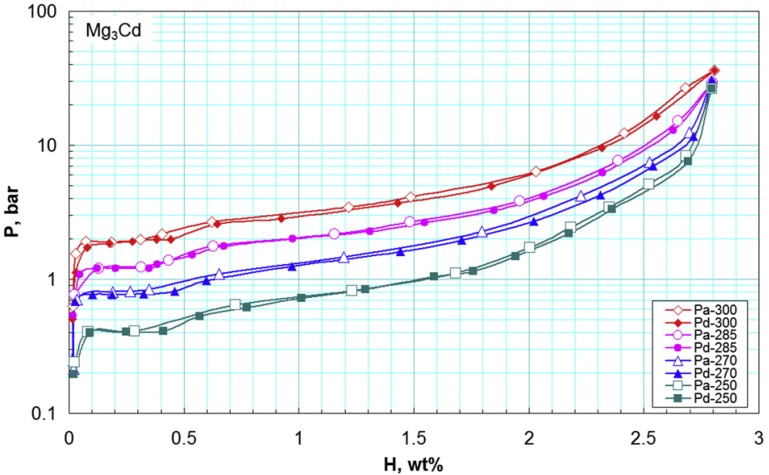
Pressure-composition-temperature curves of the Mg_3_Cd alloy in the temperature range 250–300 °C [52] (Reprinted with permission by T. Nejat Veziroglu, the founding editor-in-chief of IJHE).

Cd is the only element that can form a complete mutual solid solution with Mg under equilibrium conditions. Skripnyuk *et al.* [52] prepared the Mg_3_Cd alloy by ball milling. As shown in Figure 5, no measurable pressure hysteresis was observed from the PCI, and there is a short horizontal section in the pressure plateau between 0.1 wt % and 0.3 wt % of H_2_ corresponding to pure Mg. The subsequent pressure plateau was sloped and the formation enthalpies of the hydride calculated by the van’t Hoff equation were 76.3 ± 1, 65.2 ± 1, 65.2 ± 2, and 65.5 ± 3 kJ/mol for the alloys containing 0.25, 1.55, 1.85, and 2.00 wt % hydrogen, respectively.

Based on the above results, alloying Mg with some elements can tune the thermodynamics of MgH_2_. However, some limits cannot be ignored: the hydrogen capacity decreases sharply after alloying, and the reaction is often irreversible because of the bonds between Mg and the other element breaking during the process of hydrogenation.

### 4.2. Nanostructuring

Nanostructuring can not only enhance the kinetics of Mg-based alloys, as mentioned in the previous section, it can also destabilize the thermodynamics of MgH_2_. Wagemans *et al.* [53] investigated MgH_2_ using quantum mechanical calculations and found that nanosized MgH_2_ clusters have much lower desorption enthalpy (63 kJ/mol) than bulk MgH_2_, enabling hydrogen desorption at lower temperatures from nanoclusters than from the bulk. The desorption temperature of MgH_2_ clusters (size of ~0.9 nm) is only 200 °C.

The surface energy contributes significantly to the overall energy for the nanoparticles. Taking into account the surface energy, the total energy change upon dehydrogenation of the metal hydride will depend on the particle radius *r*, and can be expressed as [54,55]

(1) $$\Delta G=\Delta G_{0}+RT\ln\frac{p}{p_{0}}+\frac{3V_{M}\Delta_{M\to MH2}(\gamma ,r)}{r}$$

with the volume-adjusted surface energy difference ∆*_M→MH2_* given by [55]

(2) $$3V_{M}\Delta_{M\to MH2}(\gamma ,r)=\left[\gamma_{MH2}(r){\left(\frac{V_{MH2}}{V_{m}}\right)}^{\frac{2}{3}}-\gamma_{M}(r)\right]+E_{ads}$$

**Figure 6:**
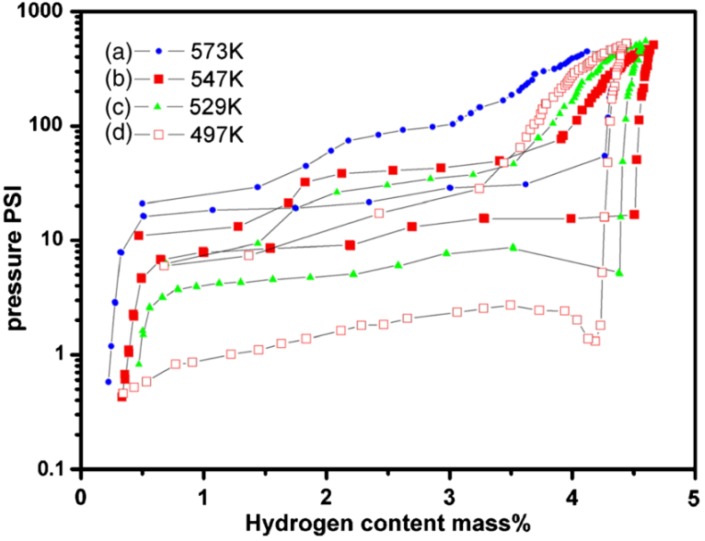
Pressure-composition isotherm curves of the Mg_2.9_Ni thin film measured at different temperatures: (**a**) 573 K; (**b**) 547 K; (**c**) 529 K; and (**d**) 497 K (Reprinted with permission from [56]. Copyright 2007 AIP Publishing LLC).

Because the surface energies might change during hydrogenation, the extra term *E_ads_* is included. *γ_MH_*_2_ and γ_M_ are the surface energies of the hydride and metal. If *γ_MH_*_2_ > *γ_M_*, decreasing the size of the nanoparticles will lower the stability of the hydride. Based on Equations (1) and (2), the formation enthalpy of sphere MgH_2_ with radii smaller than 5 nm will decrease 20% compared with that of bulk MgH_2_ in the same size. The high-density grain boundary in nanocrystalline Mg-based hydrides has a similar effect. Ouyang *et al.* [56] prepared the Mg_2.9_Ni film with a preferential orientated nanocrystalline structure via magnetron sputtering. The PCI curves of the Mg_2.9_Ni film are shown in Figure 6, and the lowest desorption temperature of this film was 497 K, which is much lower than that for bulk Mg with conventional grain size. This is because the interfacial energy of nanocrystalline Mg/Mg_2_Ni is greatly increased, which can decrease the energy barrier for the formation of new phases. They used a simplified model to estimate the extra energy stored in the interface. By including the extra stored free energy, the formation enthalpies of Mg_2_NiH_4_ and MgH_2_ decreased to −59.5 and −69.5 kJ/mol, respectively, and the reaction temperatures of Mg_2_NiH_4_ and MgH_2_ decreased to 487.7 and 514.8 K, respectively, and are close to their experimental values. In addition, hydrogen first desorbs from Mg_2_NiH_4_, and then hydrogen desorption from MgH_2_ becomes easier because of the stress caused by neighboring Mg_2_Ni decomposed from Mg_2_NiH_4_.

Lu *et al.* [57] prepared a nanostructured MgH_2_–0.1TiH_2_ material by ultrahigh-energy-high-pressure ball milling. The grain size of the powder after milling was 5–10 nm with a uniform distribution of TiH_2_ among the MgH_2_ particles. Both the nanosize and the addition of TiH_2_ contributed to the improvement of the kinetics and thermodynamics. The ΔH value for the dehydrogenation of MgH_2_–0.1TiH_2_ was 68 kJ/mol, which is lower than that of bulk MgH_2_.

**Figure 7:**
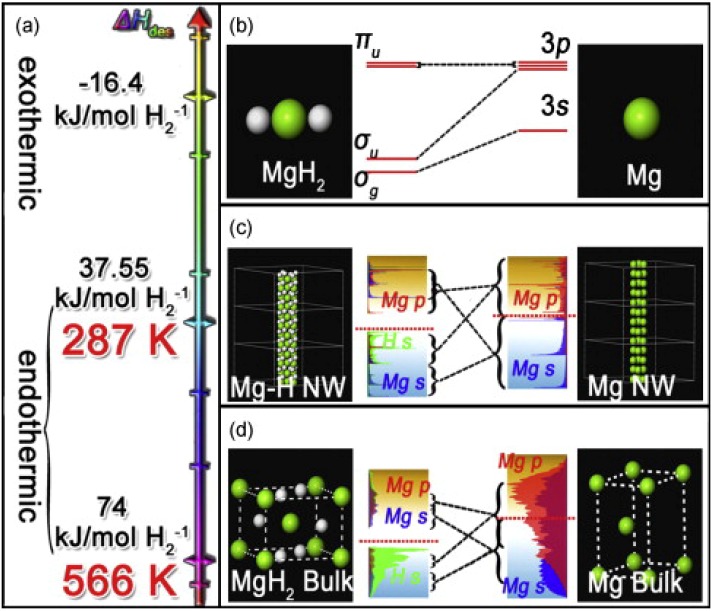
Significant difference of thermodynamic properties (**a**) and electronic structures among molecule (**b**); nanowire (**c**) and bulk (**d**) of Mg/MgH_2_ (Reprinted with permission from [59]. Copyright 2009 Elsevier).

Chen *et al.* [58] produced Mg nanowires by a vapor-transport method using commercial Mg powder. The diameters of three Mg nanowires were 30–50 nm, 80–100 nm, and 150–170 nm. Both the kinetics and thermodynamics of the Mg nanowires were better than those of bulk Mg/MgH_2_. The activation energies of hydriding/dehydriding were 33.5/38.8, 38.7/46.5 and 70.3/81.1 kJ/mol, and the ΔH’s for dehydriding of the hydride samples were 65.3, 65.9, and 67.2 kJ/mol. Moreover, as shown in Figure 7 [59], first-principles DFT calculations gave the dehydrogenation enthalpies for the MgH_2_ bulk, nanowires, and single molecule as 74.0, 37.6, and −16.4 kJ/mol, respectively. These results indicate that decreasing the diameter leads to thermodynamic destabilization of MgH_2_ nanowires, and that hydrogen desorption is possible at room temperature for MgH_2_ nanowire of a diameter of 0.85 nm [59]. However, the nanowires could not maintain their structure after several hydrogenation/dehydrogenation cycles.

To stabilize the Mg/MgH_2_ at the nano-scale, a recently developed strategy is to confine Mg/MgH_2_ in a scaffold or matrix. Konarova *et al.* [60] synthesized MgH_2_ particles by wet impregnation within the pores of the mesoporous materials SBA15 and CMK3. The thermal desorption behavior of MgH_2_/CMK3 compounds were studied by varying the MgH_2_ loading amount in CMK3. MgH_2_/CMK3 compounds with 20 wt % loading released hydrogen at a temperature of 253 °C. Its corresponding decomposition enthalpy was 52.38 ± 2.16 kJ/mol. This means that there is a substantial destabilization of the thermodynamics of the Mg-MgH_2_ reaction by nanoconfinement. Fichtner *et al.* [15] prepared MgH_2_ nanoparticles with a size of less than 3 nm by hydrogenation of Bu_2_Mg inside the pores of a carbon scaffold. Because the particles were small and the interaction between the particle and the support created strain in the nanoparticles, a significant decrease of the reaction enthalpy (63.8 ± 0.5 kJ/mol) and entropy (117.2 ± 0.8 J/mol) was obtained compared with the bulk materials. Although the reaction enthalpy greatly decreased, the decrease of the desorption temperature was only 11 °C because of the countereffect of the decreased reaction entropy. This result demonstrated that significant entropy change can occur, even though previous studies considered entropy to be a constant valve of about 130 J/(mol·K). Buckley *et al.* [16] used a mechanochemical method to synthesize MgH_2_ nanoparticles embedded in a LiCl salt matrix. When the size of MgH_2_ nanoparticles was ~7 nm, the decomposition reaction enthalpy decreased from ∆H_bulk_ = 74.06 ± 0.42 kJ/mol to ΔH_nano_ = 71.22 ± 0.49 kJ/mol. However, the reaction entropy also decreased from ∆S_bulk_ = 133.4 ± 0.7 J/(mol·K) to ∆S_nano_ = 129.6 ± 0.8 J/(mol·K). Thus, the desorption temperature of MgH_2_ nanoparticles decreased from 281.8 ± 2.2 °C (bulk MgH_2_) to 276.2 ± 2.4 °C, which is less than expected from the enthalpy change due to the decrease in ∆S.

In summary, nanostructuring can improve both the kinetics and thermodynamics of Mg-based hydrogen storage alloys. However, preparing materials with a sufficiently small dimension and maintaining the nanostructure during the cycles of hydrogenation/dehydrogenation is still difficult. In addition, size effects on a change in ∆S need to be further investigated.

### 4.3. Changing Reaction Pathway

**Figure 8:**
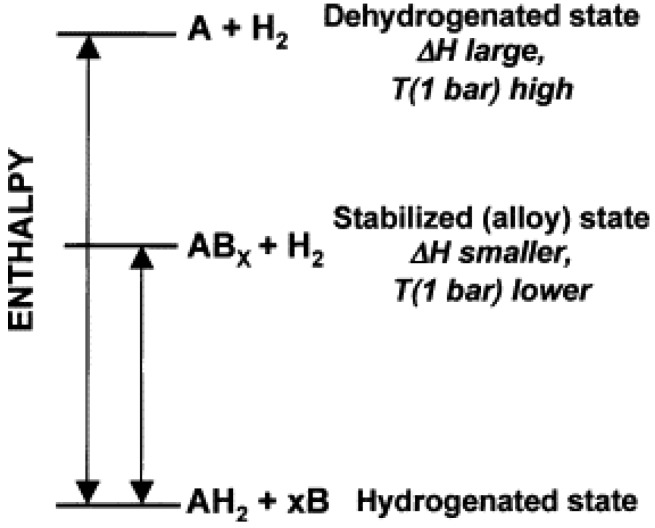
Generalized enthalpy diagram illustrating destabilization by alloy formation upon dehydrogenation (Reprinted with permission from [61]. Copyright 2007 Elsevier).

Figure 8 shows that the hydride AH_2_ can be destabilized by adding a reactive additive B. Without B, AH_2_ decomposes into A and H_2_ with a large enthalpy. In contrast, H_2_ and stable AB*_x_* form upon dehydrogenation after adding B, thus decreasing the reaction enthalpy, which raises the plateau pressure and decreases the dehydrogenation temperature [61].

A specific example of changing the reaction pathway to destabilize the thermodynamics of Mg/MgH_2_ is to add Si to create the following reaction [62]:

(3) $$MgH_{2}+Si\to Mg_{2}Si+2H_{2}$$

The desorption enthalpy decreased to 36.4 kJ/mol due to the formation of Mg_2_Si in Equation (3). However, this system suffers from poor reversibility and large capacity loss. Similarly, Walker *et al.* [63] milled MgH_2_ with Ge under an Ar atmosphere (Equation (4)). The desorption temperature of MgH_2_/Ge dramatically decreased to 130 °C. Unfortunately, the desorption product Mg_2_Ge could not absorb hydrogen to form MgH_2_ and Ge.

(4) $$MgH_{2}+Ge\to Mg_{2}Ge+2H_{2}$$

Obviously, the above two systems are irreversible. In addition, the additives Si and Ge cannot form hydrides, so the hydrogen capacity would be partially lost. However, using hydrides as additives can minimize this loss. Based on this, Vajo *et al.* [64] milled MgH_2_ with LiBH_4_. The dehydrogenation reaction can be expressed as

(5) $$MgH_{2}+2LiBH_{4}↔2LiH+MgB_{2}+4H_{2}$$

This reaction has a relatively small enthalpy of ΔH = 46 kJ/mol. Both MgH_2_ and LiBH_4_ are destabilized by the formation of MgB_2_. However, MgB_2_ is too stable to make the reaction reversible.

Al is also a reactive additive for MgH_2_. The reaction occurs via two steps [65]:

(6) $$2MgH_{2}+3Al↔Mg_{2}Al_{3}+2H_{2}$$

(7) $$9MgH_{2}+4Mg_{2}Al_{3}↔Mg_{17}Al_{12}+9H_{2}$$

For the first step (Equation (6)), the reaction enthalpy is approximately 62.7 kJ/mol. Although the whole dehydriding reaction enthalpy is 77.7 kJ/mol, which is slightly higher than that of pure MgH_2_, the whole reaction entropy increased from 139 to 144 J/(mol·K). Therefore, the desorption temperature of MgH_2_-Al would be slightly lower than pure MgH_2_ [66]. The system can reversibly store 4.4 wt % H_2_.

By mechanical alloying, a reversible hydriding/dehydriding reaction for Mg(In) solid solution was developed by Zhong *et al.* [67]. At temperatures above 573 K, the path of the hydriding/dehydriding reaction for Mg(In) solid solution is as follows:

(8) $$Mg\left(In\right)+H_{2}↔MgH_{2}+\beta$$

In the hydriding reaction, Mg(In) solid solution transforms to MgH_2_ and MgIn (β phase), while in the dehydriding reaction, MgH_2_ and MgIn can fully transform back to Mg(In). The enthalpy of this reaction is obviously less than pure Mg, and the plateau pressure of dehydriding is significantly higher. The decrease of the desorption enthalpy is related to the In content in the Mg(In) solid solution, and the enthalpy decreases from 78 kJ/mol for pure Mg to 68 kJ/mol for Mg_9__5_In_5_ with a hydrogen storage capacity of greater than 5 wt %.

Destabilizing the thermodynamics of Mg-based alloys by changing the reaction pathway has the common disadvantage that the reaction is not completely reversible. Nevertheless, it provides a new route to tune the thermodynamics of Mg/MgH_2_. It is worth noting that Mg(In) solid solution is completely reversible after dehydrogenation and the thermodynamic hydrogen sorption properties are altered. However, In is too expensive to be used on a large scale and the decrease of the enthalpy is not sufficient to make it cost efficient. Future research should focus on replacing In with cheaper elements, to further decrease the enthalpy and increase the kinetics.

### 4.4. Other Methods

Compared with bulk and powder materials, thin films often exhibit different properties. Higuchi *et al.* [68] investigated the hydrogen storage properties of nanocomposite three-layered Pd(50 nm)/Mg(*x* nm)/Pd(50 nm) films. With increasing thickness of the Mg film (*x*), the temperature corresponding to the maximum dehydrogenation rate decreased from 192 °C at *x* = 25 nm to 87 °C at *x* = 800 nm. This improvement can be explained by the cooperative phenomenon due to the elastic interaction between nanostructured Mg and Pd layers. The Pd/Mg/Pd film with a thick Mg layer would peel off from the substrate upon hydrogen uptake. For dehydrogenation, hydrogen in the up and down Pd films desorbs first, then the compression stress is induced on the up and down surfaces of the middle Mg film. As a result, hydrogen in the Mg film becomes unstable and leads to low temperature dehydrogenation. While Pd/Mg/Pd with a thin Mg layer does not peel off from the substrate upon hydrogenation, and Mg exhibits a martensite-like transformation, resulting in only a small compression stress on the Mg film plane leading to the weak cooperative interaction.

**Figure 9:**
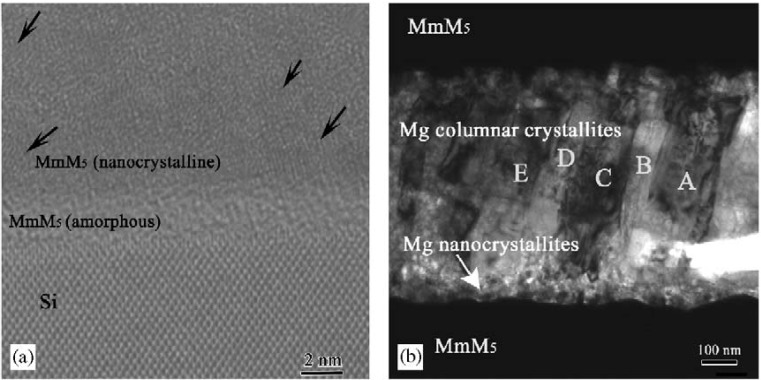
HRTEM and TEM images of the cross-sections of the (**a**) MmM_5_ layer and (**b**) Mg layer in the Mg/MmM_5_ multi-layer film [69,70]. (Reprinted with permission from [69]. Copyright 2004 John Wiley and Sons. And reprinted with permission by T. Nejat Veziroglu, the founding editor-in-chief of IJHE.)

To improve the hydrogenation properties of Mg, Wang and Ouyang et al. prepared Mg–Ni thin films, and Mg/MmM*_x_* and Mg-Ni/MmM_5_ multi-layer films [69,70,71,72,73,74]. They prepared the Mg/MmM_5_ multi-layer film via magnetron sputtering, and X-ray diffraction (XRD) analysis showed that most of the Mg was hydrogenated and dehydrogenated at about 523 K, which is about 100 K lower than the dehydrogenation temperature of pure Mg film. The microstructure of the multi-layer film was investigated in detail (Figure 9) [69,70]. The MmM_5_ layer is composed of two regions: one is an amorphous region approximately 4 nm thick at the bottom of the layer, and the other is a nanocrystalline region on top of the amorphous region. The Mg layer is also composed of two regions: a randomly orientated nanocrystalline region 50 nm thick at the bottom of the layer, and a columnar crystallite region on top of the nanocrystalline region. Most of the columnar crystallites have their (001) directions parallel to the growth direction. All of the interfaces between different layers are clean without interface phases. The hydrogenation properties of Mg can be improved when the MmM_5_ layer is inserted between the nanocrystalline Mg layers. A remarkable decrease of the hydrogenation/dehydrogenation temperature was achieved in the Mg/Mm–Ni multi-layer film prepared by evaporation deposition. Mg can be fully hydrogenated at 423 K and the hydrogen desorbs at 473 K. The decrease in hydrogenation/dehydrogenation temperature can be attributed to the catalytic roles of the Nd(La)Ni_3_ and Mg_2_Ni phases [72].

Baldi *et al.* [75] reported that the thermodynamics of hydrogen absorption in Mg can be tailored through elastic constraints. The equilibrium pressures of Mg films covered with Ni or Pd are much higher than those of Mg films capped with Ti, Nb, or V. Because Ti, Nb, and V are immiscible with Mg, the Mg layer is free to expand upon hydrogen uptake. However, Ni and Pd are Mg-alloy-forming elements, and there is strong bonding between Mg and Ni or Pd. Therefore, elastic clamping exists at the interface, leading to the occurrence of hydrogenation under high plateau pressure. The effect of the clamping is greater the thinner the Mg layer, and the plateau pressure increases with decreasing Mg thickness. A 10-nm-thick Mg film has an equilibrium pressure more than 200 times higher than bulk Mg. Thus, elastic clamping offers an alternative route for tuning the thermodynamics of Mg–H.

However, Chung *et al.* [76] showed that strain is not responsible for the increase in the equilibrium pressure for Mg thin films capped with a Pd layer, because MgH_2_ was formed with near zero stress. It was the Mg–Pd alloy formed in the intermixed region at the Mg/Pd interface that increased the equilibrium pressure. The intermixing layer between the Mg and Pd layers was clearly observed by TEM and HRTEM, while the interface between Ti and Mg shows no intermixed region. The hydrogenation reaction of Mg-Pd is different from pure Mg, and can be represented by

(9) $$\left(\frac{1}{1-x}\right){\text{Mg}}_{1-x}{\text{Pd}}_{x}+{\text{H}}_{2}↔{\text{MgH}}_{2}+\left(\frac{x}{1-x}\right)\text{Pd}$$

**Figure 10:**
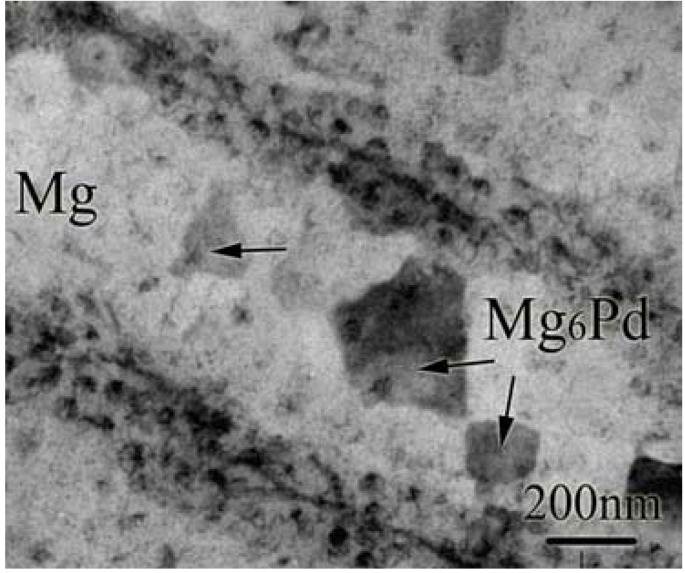
Bright-field TEM image of Mg/Pd multi-layer film activated at 473 K for 2 h under hydrogen pressure of 30 bar (Reprinted with permission from [78]. Copyright 2010 Elsevier).

The net enthalpy for the above reaction is −56 kJ/mol [77], which is smaller than that of pure Mg hydrogenation and, therefore, the equilibrium hydrogen pressure is higher. Ye *et al.* [78] prepared Mg/Pd multi-layer films by magnetron sputtering and also observed that the same dense Mg–Pd alloy Mg_6_Pd formed along the interface between Mg and Pd, and even inside the Mg layers after the films were activated at 473 K for 2 h (Figure 10). The films could absorb and desorb about 2.5 wt % hydrogen at a low temperature of 323 K. The low hydrogenation/dehydrogenation temperature can be attributed to the extra interfacial free energy in the thin films and the catalytic effect of Pd.

The ΔH values for some improved Mg-based alloys mentioned above are listed in Table 3, which illustrate that alloying, nanostructuring, and changing the reaction pathway are effective ways to alter the thermodynamics of Mg/MgH_2_.

**Table 3:** ΔH for improved Mg-based alloys in some works.

Strategies	Mg-based alloys	ΔH (kJ/mol)
Alloying	Mg_2_NiH_4_ [18]	64.5
	Mg_3_Cd–H [52]	65.2
Nanostructuring	nanostructured MgH_2_–0.1TiH_2_ [57]	68
	Mg nanowires (30–50 nm) [58]	65.3
	MgH_2_/CMK3 compounds [60]	52.38 ± 2.16
	MgH_2_/ACF composite [15]	63.8 ± 0.5
Changing the reaction pathway	MgH_2_/Si system [62]	36.4
	MgH_2_/LiBH_4_ system [64]	46
	Mg(In)–H system [67]	68

## 5. Summary and Prospects

Hydrogen storage materials and technology are the key issues in the realization of a hydrogen economy. Mg-based alloys show great promise because of their relatively high hydrogen storage capacity and abundance. Substantial progress has been made in improving the hydrogen absorption/desorption kinetics of Mg-based alloys. However, the thermodynamics of MgH_2_ are still a big challenge for the practical application of Mg-based hydrogen storage alloys.

Alloying, nanostructuring, and changing the reaction pathway can effectively destabilize MgH_2_. However, other problems arise, such as the loss of hydrogen storage capacity, the poor stability of the nanostructure, and the irreversibility of the reaction. The hydrogen capacity of MgH_2_ is close to the value of the ultimate DOE target (7.5 wt %), leaving little room to improve the thermodynamics by alloying [79]. Mixing Mg-based alloys with a compound that is itself a hydride is a possibility. Moreover, it is difficult to prepare Mg-based nanostructures with controlled size, and it is therefore necessary to develop new strategies to produce ideal nano-Mg/MgH_2_. It is worth noting that the Mg(In) solid solution can be reversibly formed by dehydriding its hydrogenated products, and has a lower reaction enthalpy than pure Mg. However, Mg(In) has poor kinetics. Finding a reversible system with a high hydrogen capacity and excellent kinetics, and then effectively tailoring the reaction enthalpy of the system, would be a solution to solve the thermodynamic tuning of Mg-based hydrogen storage alloys. There are still many challenges in this field and much work needs to be done to realize the hydrogen economy of the future.
